# Psychological interventions to promote self-forgiveness: a systematic review

**DOI:** 10.1186/s40359-024-01671-3

**Published:** 2024-05-09

**Authors:** A. Vismaya, Aswathy Gopi, John Romate, Eslavath Rajkumar

**Affiliations:** 1 Department of Psychology, Central University of Karnataka, 585367 Kalaburagi, India; 2 Department of Liberal Arts, Indian Institute of Technology Bhilai, 492015 Chhattisgarh, India

**Keywords:** Self-forgiveness, Intervention, Systematic review

## Abstract

**Background:**

Being able to forgive one’s own wrongdoings improves the health and well-being of a person. People find it difficult to forgive themselves due to different reasons. It is essential to enhance the ability to accept one’s deeds and thereby enhance self-forgiveness. The current systematic review’s objective is to comprehend the application and efficiency of numerous interventions that improve self-forgiveness.

**Methods:**

The search was done on electronic databases such as PubMed, ERIC, Web of Science, PsycNet, Science Direct, and Google Scholar. The initial search yielded 399 articles. After the duplicate removal, 19 articles met the eligibility criteria. Two studies were identified through related references. Thus, 21 articles were finalized for review. The study adhered to the PRISMA recommendations for systematic reviews.

**Results:**

The 21 finalized articles varied in method, participants, research design, duration, measurement tools used, and observed outcomes. Thirteen of the 21 finalized articles followed interventions specifically designed to promote self-forgiveness. Interventions are seen to be applied at both individual and group levels.

**Conclusion:**

The review categorizes the interventions into self-directed and group. The self-directed interventions, notably those based on Enright’s process model, demonstrate its efficiency in nurturing self-forgiveness. Self-forgiveness interventions are also found to be effective in promoting other positive psychological and clinical variables. Further implications and future research avenues are outlined.

**Supplementary Information:**

The online version contains supplementary material available at 10.1186/s40359-024-01671-3.

## Background of the study

Forgiveness as a core positive psychological and moral construct has gained recent research attention. It is defined as a “complex affective, cognitive, and behavioral phenomena in which negative affect and judgment toward one’s offender are reduced, not by denying one’s right to such affect but by viewing the offender with compassion, benevolence, and love” [1].Enright [2] described forgiveness in terms of a triad including forgiving others, obtaining forgiveness from others, and self-forgiveness. Self-forgiveness is a relatively recent and moderately explored psychological construct [3]. It has been considered within the broader framework of self-compassion as a higher-level overarching construct [4]. Despite this, it has historically and presently been regarded as one among several dimensions constituting the broader construct of forgiveness [4]. Self-forgiveness shares some similarities and differences with the forgiveness of others. Like interpersonal forgiveness, self-forgiveness can be unconditional, irrespective of the essence of the transgression [5]. Both entail the release of resentment, responding to specific events perceived as offensive to oneself or others [5]. On the other hand, self-forgiveness may rely less on the behavior of others, while forgiving others is enabled when the offender apologizes or shows regret [6]. An individual who experiences difficulties in forgiving oneself internalizes his/her negative emotions, while those who have difficulty forgiving others externalize their negative affect [7].

Various conceptualizations exist for the construct of self-forgiveness. For instance, considered as a moral virtue, it is defined as “people, on rationally determining that they have offended themselves by violating their sense of justice, self-forgive when they willfully abandon self-resentment and related responses (which begin as natural reactions when the violation of justice is acknowledged but can turn into toxic self-loathing) and endeavor to respond to themselves based on the moral principle of beneficence, which may include compassion, unconditional worth, generosity and moral love” [8]. Alternative definitions of self-forgiveness have been proposed by many scholars, such as “a set of motivational changes whereby one becomes decreasingly motivated to avoid stimuli associated with the offense, decreasingly motivated to retaliate against the self, and increasingly motivated to act benevolently towards the self” [9]. When the former defines self-forgiveness from a morality perspective, the latter describes it as a behavior or behavioral motivation. An alternative conceptualization pertains to the behavioral dimension in the process of self-forgiveness, which is “an emotion-focused coping strategy that involves reducing negative and increasing positive thoughts, emotions, motivations and behaviors regarding oneself” [10]. A comparatively broader definition recognizes self-forgiveness as “the act of generosity and kindness toward the self following self-perceived inappropriate action” [11]. The dual-process model of self-forgiveness defines “self-forgiveness as a moral repair strategy in which perpetrators (a) orient toward positive values by making a decision to accept responsibility for wrongdoing and align their behavior with positive values in the future as well as (b) restore esteem by replacing self-condemning emotions with self-affirming emotions” [12, 13].

Even though there has been tremendous research on forgiveness, relatively little is known about forgiving one’s own mistakes. Existing evidence suggests that the practice of self-forgiveness can help a lot of condemnation due to various offenses [14]. Self-forgiveness improves psychological health, including life satisfaction [15], self-esteem [16], emotional stability [17], and perceived quality of life [18]. Recent research reveals that self-compassion and self-esteem significantly impact the nature and extent of self-forgiveness [19]. It is also evident that interpersonal forgiveness, self-esteem, and self-forgiveness are all significantly correlated with subjective well-being [20]. Additionally, individuals who are more self-forgiving also tend to engage in more fulfilling interpersonal interactions [21]. Lower life satisfaction and self-esteem, as well as higher neuroticism, depression, anxiety, and anger, are all correlated with a lack of self-forgiveness [10]. Authentically forgiving oneself is one of the best ways to overcome these negative thoughts and feelings [22].

The abstract nature of self-forgiveness hinders its promotion, making it hard to enhance or cultivate [2]. Besides, Holmgren [23] suggests that the development of self-forgiveness encompasses three major components. Firstly, there needs to be an acknowledged objective wrongdoing committed by the individual, along with a genuine recognition of it as wrongdoing that causes a guilty feeling. The second element involves the individual’s ability to let go of the grudge and guilt directed towards oneself, thereby initiating the process of self-forgiveness. Lastly, self-acceptance plays a significant role; the individual must fully accept oneself, acknowledge their imperfections, and demonstrate self-compassion to fully achieve self-forgiveness [23]. Moreover, based on human experiences, most people are harder on themselves than others, making it difficult to reconcile themselves [2]. Psychological defense mechanisms, such as rationalization and moral disengagements, are employed in response to threats to self-regard or moral integrity [24, 25]. These mechanisms collectively form a psychological immune system, which shields individuals from the negative impact of transgressions by preserving optimistic self-perceptions [26]. Pseudo self-forgiveness is the process that involves offenders using these defenses to attain a positive self-regard following wrongdoing, essentially reconstructing the cognitive interpretation of their actions to mitigate emotional distress [9, 16, 27, 28]. Unlike genuine self-forgiveness, pseudo self-forgiveness lacks true acknowledgment of wrongdoing [29]. The latter is characterized as a cognitive adaptation aimed at reducing emotional strain arising from moral transgressions [30].

However, practicing forgiveness towards oneself is comparatively more effortful than forgiving others [5, 31, 32]. The extent to which a transgressor forgives oneself may be influenced by the severity of the offense, particularly in relation to its consequences [9]. Likewise, in a therapeutic context, dealing with clients who require self-forgiveness is challenging since any mistake in decision-making can lead to self-hurt or self-harming behavior [33]. Moreover, unless the self-transgression in the client is not managed correctly, it may become severe and eventually lead to depression and suicide [33].

Despite these challenges, promoting self-forgiveness is essential in interpersonal and intrapersonal contexts. In an interpersonal context, the person commits an objective wrong to another person, which induces shame or guilt in the wrongdoer [34]. An intriguing aspect of self-forgiveness is its potential to enhance interpersonal relationships. The same study reported that in an intrapersonal context, the person does wrong to oneself, such as hurting oneself verbally or physically and then having a regretful negative feeling towards oneself [34].

Systematic reviews in the area of self-forgiveness are limited. A study that explores the nature and relationship between self-compassion, self-harm or suicidal ideation is the only systematic review that is been conducted in this area [35]. Just like the interventions in forgiveness of others, numerous interventions have been employed to enhance self-forgiveness. However, no systematic review has been conducted to analyze various interventions that enhance self-forgiveness. Although a related review of self-forgiveness exists [36], the present study attempts to bring an in-depth analysis of the characteristics of interventions and outcomes. Hence, the aim of the systematic review includes (a) a narrative or descriptive synthesis of existing self-forgiveness interventions based on their characteristics and effectiveness and (b) to comprehensively present various positive psychological, clinical, and physiological outcomes of the interventions that promote self-forgiveness.

## Methods

The guidelines for Preferred Reporting Items for Systematic Reviews and Meta-Analyses (PRISMA) were followed for the current systematic review [37].

### Eligibility criteria

Quantitative and qualitative studies published in the English language with a focus on promoting self-forgiveness across diverse populations were included. No limits were placed on gender, age, ethnicity of the participants, and year of publication due to the limited number of studies in the area. Whereas, review papers, book chapters, conference proceedings, and abstracts were excluded.

### Information sources and search strategy

Two authors independently searched PubMed, Web of Science, PsycNet, ScienceDirect, ERIC, and Google Scholar in January 2024. The broad keywords such as “self-forgiveness” OR “self forgiveness” were used due to limited number of studies in the area. These search terms were employed in each online database according to their search strategy. For instance, search strategy used in PubMed: (“self-forgiveness"[Title/Abstract]) OR (“self forgiveness"[Title/Abstract]), Web of Science: (TS=(“self-forgiveness”)) OR TS=(“self forgiveness”), PsycNet: Abstract: “self forgiveness” OR Abstract: “self forgiveness”, ScienceDirect: Title, abstract, keywords: “self-forgiveness” OR “self forgiveness”, ERIC: “self-forgiveness” OR “self forgiveness”.

### Selection process

The relevant articles yielded from databases were exported to Zotero reference management software. After the removal of duplicates, the remaining studies were screened for title/ abstract by two reviewers. Studies that were found ineligible at this stage were removed. Full texts were retrieved for the studies that met eligibility criteria. Consequently, the same authors independently performed the full-text analysis. Any disagreements between the two reviewers during the selection process were resolved through consultation with the third reviewer.

### Data collection process

After the full-text screening, significant information was extracted from eligible reports, including the name of the author(s) and year of publication, country, study design, characteristics of participants (sample and sample size), intervention promoting self-forgiveness, duration of intervention, and study outcomes.

### Quality assessment and data synthesis

The risk of bias in the included studies was assessed by two reviewers using JBI critical appraisal tools for randomized controlled trials (RCTs) [36], quasi-experimental studies [38] and qualitative studies [39]. The total “Yes” score ranges from 0 to 13 for randomized controlled studies, 0 to 9 for quasi-experimental studies, and 0 to 10 for qualitative studies. For studies with randomized controlled trials, a score of 1–4 indicates low quality, 5–8 medium quality, and 9–13 high quality. For the quasi-experimental reports, a quality score of ≥ 6 was considered. Regarding qualitative studies, 0–3 indicates high risk, 4–7 indicates moderate risk, and 8–10 indicates low risk. The checklist for randomized controlled trials included proper randomization baseline similarity and concealment of treatment being provided to assigned groups. The criteria for quasi-experimental reports included the presence of a control group, baseline similarity, and reliability of the measures used. The checklist for qualitative studies assessed the philosophical perspectives, methodological approaches, and ethical considerations in the included studies. The extracted evidence from eligible studies was narratively synthesized and presented descriptively.

## Results

### Study selection

**Fig. 1:**
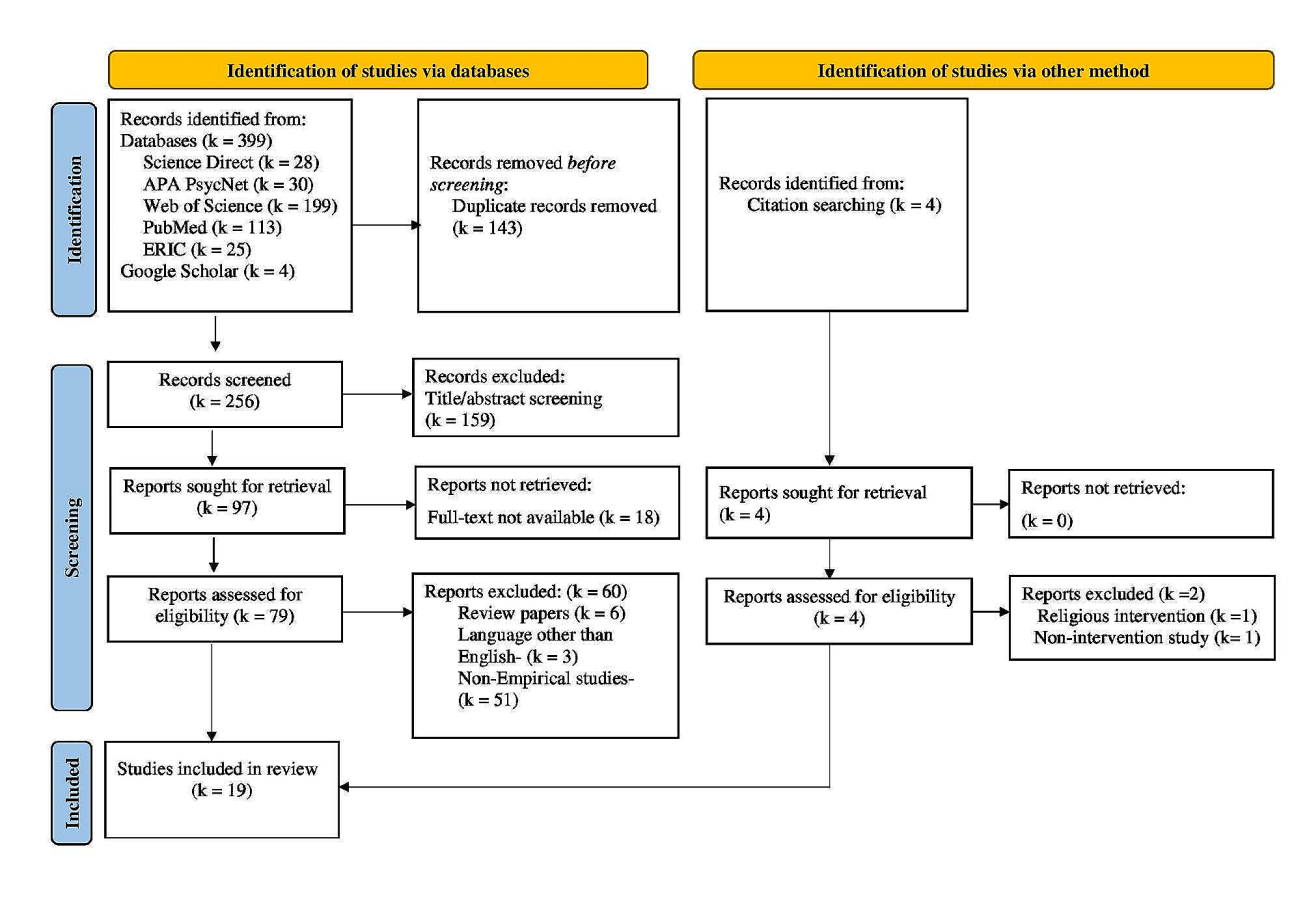
PRISMA flow diagram

The systematic searches across the databases returned 399 records, including 30 studies from APA PsycNet, 28 from ScienceDirect, 113 from PubMed, 199 from Web of Science, 25 from ERIC, and a manual search from Google Scholar yielded four reports (see Fig. 1). After removing the duplicates, 256 records were screened based on title and abstract. This phase removed titles/abstracts that did not meet the eligibility criteria (k = 159). Out of 97 studies sought for full-text, 18 were unavailable. Thus, a full-text analysis has been done for the remaining 79 studies. Subsequently, 60 reports were eliminated due to various reasons, such as review papers (k = 6), non-English studies (k = 3), and non-empirical studies (k = 51). After excluding ineligible reports, 19 studies remained within the purview of analysis. Further, two studies were identified through a related citation search. Thus, the final analysis included 21 studies focusing on psychological interventions that enhance self-forgiveness.

### Study characteristics

The included studies were conducted in the USA (k = 14), Korea (k = 1), Indonesia (k = 1), Istanbul (k = 1), Australia (k = 1), New Zealand (k = 1), UK (k = 1), and North America (k = 1) from 1997 to 2023. Among the 21 articles analyzed, 18 studies were in general populations. Two studies were in clinical populations, such as cancer patients [35] and participants with eating disorders [40], and the remaining one included people undergoing alcohol abuse treatment programs [41]. The finalized articles were heterogeneous regarding research design, forms of interventions, and outcomes. Hence, the data was synthesized narratively or descriptively. Details of the included studies are shown in Table 1. Based on the review’s objectives, the data analysis is restricted to identifying interventions to facilitate self-forgiveness and summarizing their impact on clinical and positive psychological outcomes.

**Table 1:** Summary of studies included in the review

Sl. No.	Study & Year	Country	Study design	Population& age	Sample size	Intervention	Duration	Measured outcome(s)	Quality score	
1	Bell et al. (2017) [42]	USA	Randomized pretest-post-test experimental design	Undergraduate students	93 (E=50, C= 43)	Workbook-based intervention to enhance self-forgiveness	An average 70-minute single-sitting intervention	Dispositional self-forgiveness, State self-forgiveness, Acceptance of responsibility, Willingness to make reparations	8	
2	Campana (2010) [43]	USA	Randomized waitlist control, experimental design	Adult women college students of mean age 18.89	209	Workbook intervention based on Worthington’s REACH model	15 sessions, which participants completed at their own pace in 2 weeks.	Anger, shame, guilt, attachment style, trait forgiveness, unforgiveness, compassion, self-esteem	11	
3	Cornish et al. (2015) [14]	USA	Waitlist-controlled test-retest design	Adults	26	Emotion-focused individual counselling intervention	Eight weekly 50-minute sessions	Self-condemnation, Self-forgiveness, psychological distress, Self-compassion	12	
4	Coyle & Enright (1997) [44]	USA	Randomized waitlist control trial	Men hurt by abortion decision of partner. Mean age 28	10	Process model developed by Enright and Human Development study group.	12 weekly sessions, each for approximately 90 min	State anger, grief, interpersonal forgiveness, and self-forgiveness.	10	
5	Eaton et al. (2020) [45]	USA	Pretest posttest design	Participants from the university a mean age of 27.7	24	Guided imagery intervention based on Internal Family System based Self-forgiveness intervention	Seven epochs, each for five minutes	HRV, perceived stress, anger, self-forgiveness, forgiveness towards others, situational forgiveness, dispositional forgiveness	6	
6	Exline et al. (2011) [46]	USA	Randomised 2 × 2 design	Undergraduate students of mean age 19.3	172	Exercise carried out in a lab to encourage reparatory behaviors and self-forgiveness.	Not mentioned specifically	Reparatory behaviors (apology, remorse, self-condemnation, positive self-attitude) and self-forgiveness	9	
7	Griffin et al. (2015) [13]	USA	Wait-list intervention design	Undergraduate students	140 (Immediate treatment condition= 65, wait-list control condition= 75)	Workbook intervention based on the conceptualization of Worthington’s (2013) for a responsible self-forgiveness	Six hours	Genuine self-forgiveness, self-forgiveness feelings and actions, self-forgiving beliefs, guilt, shame	10	
8	Hanna (2012) [47]	UK	URN randomization design	Ex-substance abusers (44.28)	31	Secular forgiveness program (Psychoeducation)	10 sessions, each with 90 min	Self-forgiveness, interpersonal forgiveness and well-being, depression, anxiety, shame.	11	
9	Jo and An. (2018) [48]	Korea	Non-equivalent control group pretest-post-test design	Adults (65 and above) from 2 nursing homes	47 (E=24, C=23)	The group reminiscence program	Eight weekly sessions, each session for 50 min	Self-forgiveness, life satisfaction, death anxiety	7	
10	Kahija, Y.F.L., et al. (2022) [49]	Indonesia	Quasi-experimental one-group pre-test- post-test design	Undergraduate students	9	The forgiveness meditation treatment built on Vimalaramsi (2015)	The intervention was conducted in groups, four sessions within two weeks, with 90- 150 min for each session.	Self-forgiveness, forgiveness of others, situational forgiveness	7	
11	Lander (2012) [40]	North America	Case study	Woman with an eating disorder at age 26	1	Process model of Enright and human development study group.	24 weekly sessions	Self-forgiveness	8	
12	Maguen et al. (2017) [50]	USA	Randomized control trial	Clinicians with a mean age of 61.2	33	Individual psychotherapy (Cognitive-behavioral intervention)	Six to eight sessions, each for 60 to 90 min.	PTSD, self-forgiveness, psychological distress.	10	
13	Massengale, M. et al. (2020) [51]	USA	Randomized waitlist control trial	College students age ranging from 19 to 57	107 (E= 53, C=54)	Self-directed workbook intervention, which followed Worthington’s REACH model	Two hours	Self-forgiveness, maladaptive perfectionism, well-being	10	
14	Maynard et al. (2023) [52]	New Zealand	Thematic analysis	Adults aged between 18 and 65	36	Compassion-focused group therapy	Two hours with 15 min break	Self-compassion and self-forgiveness, psychological health	8	
15	Ogunyemi et al. (2020) [53]	USA (south, west, Midwest, and Northeast)	Pretest post-test design	Medical education professionals	91	Internal family system-based guided meditation intervention.	15-minute audio meditation	Self-forgiveness, forgiving other people, and situational forgiveness	5	
16	Parlak & Gul. (2021) [54]	Istanbul	Control group, pretest-posttest quasi experimental	University students of age between 18 and 23	20	Psychodrama-oriented forgiveness flexibility group program	16-week group study with one session per week for 3 h.	Self-forgiveness, forgiving others, and situational forgiveness	7	
17	Peterson, et al. (2016) [55]	USA	Randomized pretest-post-test experimental design	Undergraduate students	462 (E= 231, C= 231)	Self-forgiveness condition prompts	Not mentioned specifically	Remorse and self-condemnation, Self-forgiveness, future responsible drinking intention.	9	
18	Scherer et al. (2011) [41]	USA	Randomized experimental group with pretest and post-test	Individuals undergoing an alcohol abuse treatment program	79 (E=41, C= 38)	Four-hour self-forgiveness intervention, by adapting Worthigton’s five-step model	Four-hour intervention conducted in three 90-minute sessions for three successive weeks	Self-forgiveness, drinking refusal, self-efficacy, guilt, and shame on alcohol-related transgression	9	
19	Toussaint et al. (2014) [35]	USA	Randomized waitlist control trial	Cancer patients and caregivers	83 (E = 45, C = 38)	Self-forgiveness education	Not mentioned specifically	Self-forgiveness, Self-acceptance, Self-improvement, Pessimism	8	
20	Woodyatt & Wenzel (2014) [56]	Australia	Randomized control trial	University students have a mean age of 21.7	97	Affirmation intervention.	Not mentioned specifically	Genuine self-forgiveness, shame, self-trust, and desire for reconciliation	12	
21	Zahorcova, et al. (2021) [57]	USA	Randomized pre-test- post-test experimental design	Grieving parents	21 (E = 11, C = 10)	Educational forgiveness intervention founded on the model of Enright (2001)	12 weekly sessions for one hour	Forgiveness, Grief, Self-forgiveness, Anger, Anxiety, Depression, Self-esteem, Hope, Meaning in life, post-traumatic growth	11	

### Risk of bias

As mentioned above, the risk of bias was assessed using the JBI critical appraisal tool. Fourteen studies were randomized controlled trials [15, 29, 58–44], five were quasi-experimental [45, 48, 53, 54, 58], and two were qualitative studies [40, 52]. All fourteen RCTs were high quality according to the appraisal tool, and all five quasi-experimental studies and two qualitative studies were eligible since they were above the cut-off quality score.

### Intervention program and procedure

This section concisely overviews the interventions and their effect on clinical and positive psychological variables across diverse populations. Depending upon the nature of the intervention, it is grouped into two broad categories: (1) self-directed interventions and (2) group interventions. Self-directed interventions are fully directed by self without any human contact or guidance, while group interventions are administered to groups of people rather than individuals.

1. Self-directed interventions.

In the present systematic review, 16 self-directed interventions were identified under five categories. Those are further categorized into five: (a) REACH model-based workbook interventions, (b) Enright’s process model-based interventions, (c) Therapeutic interventions, (d) Guided imagery interventions, and (e) Other interventions.

- REACH model-based workbook interventions.

Worthington’s REACH model [59] was incorporated in four of the 16 self-directed interventions [13, 41, 43, 51]. From the self-forgiveness point of view, REACH stands for Recalling the hurt, Empathise with oneself, Altruistically gifting oneself, Committed to the self-forgiveness process, and Hold on to the attained state [59]. Griffin et al. [13] followed a six-step theory of intervention, such as receiving divine self-forgiveness, repairing the social bond, restoring positive self-regard, rethinking the rumination, REACH model of self-forgiveness, rebuilding the self-acceptance and resolving to live with virtue. The study by Massengale and Michael [51] adopted the intervention procedure of Griffin et al. [13]. They incorporated terminology and methods tailored to address these issues, which involve educating individuals about self-appraisal issues and navigating the challenges associated with perfectionist tendencies using cognitive therapy techniques. Scherer et al. [41] introduced an additional layer to the intervention landscape by integrating motivational interviewing techniques designed to enhance motivation for change and mitigate resistance to participation, particularly within the framework of the REACH model. Their emphasis on self-discovery contrasts with direct educational methods, suggesting a nuanced approach to facilitating self-forgiveness. Further, the REACH model was found to be applied by Campana [43] in their study.

Among these four studies, the first two were conducted on college students [13, 51], the third one on individuals undergoing alcohol treatment programs [41], and the fourth among women experiencing breakups [43]. The duration of intervention also varied such as six hours [13], two hours [51], and four hours within three weeks (30 to 90 min for each session) [41] and in the fourth study [43], the participants were free to finish the 15 sections of intervention at their own pace with two weeks. The first three studies described here effectively enhanced the self-forgiveness of the participants, whereas the study by Campana [43] showed no significant change in self-forgiveness. Positive psychological outcomes other than self-forgiveness included well-being [51], drinking refusal, self-efficacy [40], compassion, self-esteem, and trait forgiveness [43]. The clinical outcomes of different studies include reduced state guilt, state shame [13, 41, 43], and anger [43].

(b) Enright’s process model-based interventions.

Four of the included studies [40, 44, 47, 57] followed Enright’s process model, a psychoeducation intervention of self-forgiveness. The intervention module has four phases. The first phase is uncovering the emotions and defense mechanisms related to the hurt situation. Second is the decision phase, in which the participant commits to forgive. Third is the work phase, during which participants explore the past of the wrongdoer and build empathy and compassion. The fourth is the deepening phase, which focuses on finding new meaning in life. For each session, the participants were asked to go through a chapter in the book based on which upcoming session will be dealt with. The populations included grieving parents [57], ex-substance abusers [47], men hurt by the abortion decision of partner [44], and woman with eating disorder [40]. The duration of the study varied, as shown by 12 weekly one-hour sessions [57], 10 weekly 90-minute sessions [47], 12 weekly 90-minute sessions [44], and 24 weekly sessions [40]. All four studies effectively enhanced the self-forgiveness of the participants. Other positive psychological outcomes observed in the studies include forgiveness towards others, self-esteem, hope, meaning in life, post-traumatic growth [57] happiness, and well-being [47]. Similarly, a significant reduction in clinical variables was observed in three of the studies, such as anxiety [47, 57], depression [44, 57], anger, grief [44, 57], and shame [47].

(c) Therapeutic intervention.

There are different therapeutic interventions in the field of Psychology. The result of this review reveals two major self-directed therapeutic interventions that enhance the self-forgiveness of the participants. In their study, Cornish and Wade [14] tested the efficacy of emotion-focused counseling on self-forgiveness. They adapted the intervention developed by Worthington [60] with certain additions regarding self-forgiveness. The intervention incorporates Emotion-Focused Therapy (EFT) and the four Rs (Responsibility, Remorse, Restoration, and Renewal) of genuine self-forgiveness. The first task of the intervention is to make the participant accept responsibility for their action through discussions with the therapist. Maguen [50] evaluated the Impact of Killing (IOK) intervention grounded in cognitive behavior therapy and trauma-focused treatment. Initial sessions aimed to destigmatize veterans’ reactions, followed by assignments focusing on self-forgiveness and cognitive behavior therapy techniques. The interventions were implemented in healthy adults [14] and combat veterans [50]. The duration of the programs was 50-minute sessions over eight weeks [15] as well as six to eight sessions lasting 60 to 90 min [50] each. Cornish and Wade [14] observed an improvement in self-compassion in the study. Both interventions resulted in a significant reduction of clinical psychological variables such as self-condemnation and psychological distress [14, 50] as well as post-traumatic stress disorder [50].

(d) Guided imagery interventions.

The result reveals the use of an Internal Family System (IFS) based guided imagery intervention in two of the studies [45, 53]. The guided imagery session comprised seven epochs, each lasting around five minutes and designed to evoke specific emotional states. Epochs included baseline, guided relaxation, second baseline, recalling the transgression, inner critique, authentic self-reflection, and concluding with the self-forgiving state. The guided meditation is implemented in university students [45] and medical education professionals [53]. The study by Eaton and Ferrari [45] enhanced dispositional self-forgiveness, state self-forgiveness, and parasympathetic responses. At the same time, the other study enhanced self-forgiveness, the forgiveness of others, and the situational forgiveness of medical education professionals [53].

(e) Other interventions.

Certain studies examined self-forgiveness development through unique interventions [28, 42, 46, 55]. Woodyat and Wenzel [28] used transgression-relevant value affirmation in their study. The participants were instructed to identify a personally significant value, articulate the reasons behind its importance, and discuss a past instance in which their behavior aligned with that value. Bell et al. [61] crafted a manual comprising three components to foster self-forgiveness: (1) promoting a prosocial and responsible attitude through solution-focused strategies and psychoeducation; (2) removing obstacles to self-forgiveness by fostering unconditioned self-acceptance and reducing shame; and (3) encouraging healthy thoughts and behaviors to facilitate planning and sustaining self-forgiveness. Exline et al. [46] conducted an experiment in which the participants were asked to list any obstacles to self-forgiveness. They were then urged to let go of any extra guilt or sentiments of self-punishment they might have had in the past. Peterson et al. [55] developed an individualized approach in which the experimental group responded to eight questions promoting self-forgiveness related to drinking-related transgression. All four studies were conducted on undergraduate students. Among the four studies, the duration of intervention was mentioned only for the study [61], a single-sitting program that lasted 70 min. Self-forgiveness was enhanced in the study by Woodyat and Wenzel [29]; self-forgiving feelings and actions were improved by the study of Bell et al. [61], but not self-forgiving beliefs. Two studies reported an increase in positive psychological variables, self-trust [28], acceptance of responsibility, and willingness to make reparations [61]. The study by Woodyat and Wenzel [28] had a clinical outcome: a reduction in the state shame of the participants. Both studies by Exline et al. [46] and Peterson et al. [55] did not result in a significant change in any of the observed outcome variables, including self-forgiveness.

(2) Group interventions.

There are five articles [51, 50, 62–64] that incorporate interventions that are implemented via the group. All these five interventions are peculiar in nature, which include a reminiscence program, psychoeducation, meditation, psychodrama, and therapy.

Parlak and Gul [54] examined a “psychodrama-oriented forgiveness flexibility group program” featuring doubling, role reversal, mirroring, cognitive rework, and ceremonial support techniques. The participant-centered intervention comprised warm-up, play, and sharing stages, during which participants observed their self-forgiveness. Another group-based intervention study [52] identified in the present review focused on the effectiveness of compassion-focused therapy. It included psychoeducation regarding the evolutionary aspects of compassion, the neuroscience of the emotion regulation system, and common obstacles in developing compassion. Further, the intervention used Compassionate Mind Training (CMT), which helps in developing a calm mind and a compassionate identity [52].

Toussaint et al. [35] implemented a group-based intervention combining self-acceptance and self-improvement. The intervention utilized workbook activities supplemented by meditation, reflection, and expressive writing. Participants were introduced to the concept and goals of self-forgiveness, followed by discussions on self-acceptance and self-improvement. The intervention concluded with a focus on maintaining commitment and achieving growth. Kahija et al. [58] investigated the efficacy of a meditation intervention. The session consisted of an introduction, relaxation technique, meditation practice, and distribution of the training kits to the participants. It ended by creating a determination in the participants’ minds. Jo and An [48] investigated the efficacy of a group reminiscence program grounded in the life review theory to enhance older adults’ self-concept. The program encompassed themes covering self-introduction, attitudes towards family, marriage, hardships, aging, preparations for death, designing present and future, and summary of thoughts.

The group-based intervention studies were implemented in diverse populations such as university students [54], adults [52], cancer patients and their caregivers [35], and adults from nursing homes [48]. The duration of each study was different. The duration of group interventions varied by 16 weekly sessions, each nearly three hours [62], two hours [64], four sessions for two weeks (each 90–150 min) [42], and eight 50-minute sessions [48]. Duration of intervention was not mentioned in a study by Toussaint et al. [35]. The study by Parlak and Gul [54] and Maynard [52] significantly enhanced the self-forgiveness of the participants. On the other hand, the study by Toussaint et al. [35] could enhance the self-forgiveness feelings and actions but not self-forgiving beliefs. Contrary to the existing findings, the study by Jo and An [48] did not enhance self-forgiveness, while the study by Kahija et al. [58] could not bring a significant change in any of the observed outcome variables. There are certain positive psychological variables that are improved through different interventions in these studies, such as forgiveness towards others and situational forgiveness [62], self-compassion [64], self-acceptance, self-improvement [51], and life satisfaction [48]. Group interventions also resulted in the significant reduction of two clinical variables, such as pessimism [35] and death anxiety [48].

## Discussion

The present systematic review synthesizes evidence on available interventions in promoting self-forgiveness through evidence from 21 studies. As per the author’s knowledge, this is the first systematic review that narratively presents these interventions based on their characteristics and outcomes. The result of the systematic review shows a diversity in interventions that promote self-forgiveness within the method followed, duration, population, and outcomes observed. Among the 21 studies that examined the effectiveness of intervention in self-forgiveness, 13 are specifically designed to enhance self-forgiveness.

Studies are categorized as self-directed and group interventions based on the types of interventions. A superiority of self-directed interventions over group interventions is seen in the results. There is a possibility of different reasons for participants to prefer self-directed interventions over group interventions. One of the major reasons is the level of shame and distress associated with disclosing one’s wrongdoing [62]. Further, the research by Lundahl et al. [63] states that programs delivered individually are superior to those delivered in groups. The present findings further categorize self-directed interventions into five: REACH model-based workbook interventions, Enright’s process model-based interventions, therapeutic interventions, guided imagery interventions, and other interventions. The REACH model [64] and Enright’s Process model [2] are two process models of forgiveness as well as self-forgiveness. This is in line with Baskin and Enright [65] and Wade and Worthington [34], who provided evidence on the role of process models in forgiveness.

Even though the usage of REACH model-based workbook interventions and Enright’s process model interventions are found to be equal in number, the ineffectiveness of REACH model to enhance self-forgiveness in the study by Campana [43] might be an indication of Enright’s process model to be better than REACH model. The result is similar in the aspect of forgiving others. For instance, Lundahl et al. [63] and Aktar and Barlow [66] state in their study that Enright’s process model outperformed the REACH model. Psychoeducation interventions offer education and therapeutic strategies that improve the quality of life of the participants and decrease the possibility of relapse [67]. The length of the intervention could also be a criterion responsible for bringing a significant output. Interventions based on Enright’s process model are comparatively lengthier than the others. Therefore, further research in different populations and large sample sizes is required to clarify the effectiveness and factors behind these results.

Other than these two models mentioned above, therapeutic and guided imagery-based meditation interventions have also been found to be effective in enhancing self-forgiveness. Concerning the therapeutic intervention by Cornish and Wade [15], exploring conflicting emotions and views about themselves through emotion-focused therapy helped the intervention to be effective [68]. The therapeutic stages, such as recognition, responsibility, expression, and recreation developed by Jacinto and Edwards [81], are yet an underexplored therapeutic model of self-forgiveness. The therapeutic interventions are applied in two populations: healthy adults and combat veterans. Further, there will be different populations that require therapeutic assistance in forgiving oneself. The IFS approach is followed in the two guided imagery-based meditation interventions, which help the participants to release the burden of past life by acting on the emotional, developmental, and cognitive dimensions of a person [69].

Group interventions are found to be comparatively lesser in number than self-directed interventions in the area of self-forgiveness. Self-condemnations occur when we disrupt our own ethical standards just to meet societal demands; it can be resolved through self-forgiveness [70]. Hence, Self-forgiveness is not only a factor that depends on and affects an individual, but also the social expectations and value set that one person is surrounded with. Due to these reasons, further studies can focus on treatments delivered in groups. Among the five group interventions analyzed, two of them could not enhance the self-forgiveness of the participants. The duration of intervention was less in the study of Kahija et al. [58] which could be a possible reason for its ineffectiveness. Coping with important negative life events to establish ego integration and to offer a coping mechanism is one of the functions of reminiscence [71]. However, the participants who were elderly people were not willing to discuss negative events of their lives. This could be a factor for the failure of intervention to show a change in self-forgiveness in the study by Jo and An [48].

Most of these studies are tested in a variety of populations. There are only two studies that are conducted in clinical populations, such as cancer patients and people with eating disorders. However, there are many other clinical populations that demand treatment for self-forgiveness. Participants affected by HIV/AIDS report low self-forgiveness and life satisfaction [72]. Fibromyalgia patients also report lower self-forgiveness scores [73]. Similarly, there are several clinical and positive psychological outcomes that can be further tested. Literature reveals that parameters such as social exclusion, internet addiction [74], hypersexual behavior [88], and chronic unhealthy behavior [75] which are negatively correlated with self-forgiveness. At the same time, positive psychological variables such as humility [76]and flourishing [77] are positively associated with self-forgiveness. Interventions emphasizing these variables can be considered in future research.

Quality assessment using JBI checklists revealed a high quality for the included studies. However, most randomized controlled trials did not provide information regarding those allocating treatment blinded to treatment assignment. In the case of quasi-experimental studies, many did not mention the comparison group and the follow-up assessments. Among the two qualitative studies, one study [52] did not provide detailed evidence of the representation of participants in the conclusion. Whereas in the other [40] ethical considerations were not adequately reported. Thus, the quality assessment of the finalized articles suggests further research to overcome these methodological concerns.

### Implications of findings

The current findings contribute to the extant literature on self-forgiveness by highlighting the predominance of self-directed interventions in promoting self-forgiveness. Also, the result emphasizes the applicability of Enright’s process model as a widely accepted approach to developing self-forgiveness. However, longitudinal studies are required to assess the long-term effects and sustainability of self-forgiveness interventions over time. Also, studies that compare the effectiveness of various intervention approaches (e.g., cognitive-behavioral therapy, psychodrama, mindfulness) to identify the most efficacious strategies to enhance self-forgiveness are critical. The findings also support clinical and non-clinical implications. Psychologists who work with individuals having self-condemnation issues due to different circumstances can apply self-forgiveness interventions. Besides, mental health professionals can integrate self-forgiveness interventions into therapeutic practices, particularly for clients struggling with guilt, shame, and self-blame associated with past transgressions or trauma. Moreover, self-forgiveness interventions are crucial in everyday life as they help to reduce negative intrapersonal and interpersonal behaviors and boost various positive aspects of psychological well-being. Encouraging self-forgiveness can facilitate personal growth and transformation, empowering individuals to move forward with renewed purpose and authenticity in their lives.

### Limitations of the study

The articles in the systematic review were confined to studies in the English language. Hence, there is a possibility of selection bias. Self-forgiveness interventions can be applied to diverse populations with large sample sizes. Variables like self-condemnation, self-compassion, and self-forgiveness, which may be highly correlated, are not emphasized in the existing interventions that warrant further attention. Future research should focus on how self-forgiveness overlaps and differs from other variables. Research is needed to identify the barriers and facilitators in the therapeutic process of self-forgiveness. Further, the feasibility and effectiveness of delivering self-forgiveness interventions through technology-based platforms, such as smartphone apps or online programs, need to be explored to increase accessibility and reach a broader audience.

## Conclusion

The systematic review provides valuable insights into interventions aimed at promoting self-forgiveness from the 21 studies. Characteristics of interventions, duration, population, and positive psychological and clinical outcomes are analyzed. Self-directed interventions, particularly those based on Enright’s process model, are efficient in fostering self-forgiveness. The findings not only enrich the existing literature on self-forgiveness but also offer practical implications for psychologists to use the interventions for the clients in need of it.

### Electronic supplementary material

Below is the link to the electronic supplementary material.

Supplementary Material 1

## Data Availability

All data generated or analyzed during this study are included in this published article [and its supplementary information files].

## Abbreviations

RCT: Randomized Controlled Trials
EFT: Emotion Focused Therapy

## References

[1] McCullough ME, Worthington EL (1995). Promoting forgiveness: a comparison of two brief psychoeducational group interventions with a waiting-list control. Couns Values.

[2] Enright RD, Freedman S. The moral development of forgiveness forgiveness therapy for those with Road rage view project forgiveness toward parents in Chinese and American families. The Influence of Filial Piety and Attribution of Responsibility View project; 1991.

[3] Vitz PC, Meade JM (2011). Self-forgiveness in psychology and psychotherapy: a critique. J Relig Health.

[4] McConnell JM (2015). A conceptual-theoretical-empirical Framework for Self-Forgiveness: implications for Research and Practice. Basic Appl Soc Psych.

[5] Enright RD. Counseling Within the Forgiveness Triad: On Forgiving, Receiving Forgiveness, and Self-Forgiveness.

[6] Darby BW, Schlenker BR (1982). Children’s reactions to apologies. J Pers Soc Psychol.

[7] Worthington EL, Lavelock C, vanOyen Witvliet C, Rye MS, Tsang JA, Toussaint L. Measures of forgiveness: Self-Report, physiological, Chemical, and behavioral indicators. Measures of personality and social psychological constructs. Elsevier Inc.; 2015. pp. 474–502.

[8] Kim JJ, Volk F, Enright RD (2022). Validating the Enright Self-Forgiveness Inventory (ESFI). Curr Psychol.

[9] Hall JH, Fincham FD (2005). Self-forgiveness: the stepchild of forgiveness research. J Soc Clin Psychol.

[10] Davis DE, Ho MY, Griffin BJ, Bell C, Hook JN, Van Tongeren DR, DeBlaere C, Worthington EL, Westbrook CJ (2015). Forgiving the self and physical and mental health correlates: a meta-analytic review. J Couns Psychol.

[11] Bryan AO, Theriault JL, Bryan CJ (2014). Self-forgiveness, posttraumatic stress, and suicide attempts among military personnel and veterans. Traumatol (Tallahass Fla).

[12] Griffin BJ, Worthington EL, Davis DE, Hook JN, Maguen S (2018). Development of the self-forgiveness dual-process scale. J Couns Psychol.

[13] Griffin BJ, Worthington EL, Lavelock CR, Greer CL, Lin Y, Davis DE, Hook JN (2015). Efficacy of a self-forgiveness workbook: a randomized controlled trial with interpersonal offenders. J Couns Psychol.

[14] Cornish MA, Wade NG (2015). A therapeutic model of self-forgiveness with intervention strategies for counselors. J Couns Dev.

[15] Thompson LY, Snyder CR, Hoffman L (2005). Dispositionol forgiveness of self, others, and situations. J Pers.

[16] Fisher ML, Exline JJ (2006). Self-forgiveness versus excusing: the roles of remorse, effort, and acceptance of responsibility. Self Identity.

[17] Walker DF. Gorsuch RL Forgiveness within the Big Five personality model.

[18] Romero C, Kalidas M, Elledge R, Chang J, Liscum KR, Friedman LC (2006). Self-forgiveness, spirituality, and psychological adjustment in women with breast cancer. J Behav Med.

[19] Pandey R, Tiwari GK, Pandey R, Mandal SP, Mudgal S, Parihar P, Rai PK, Sudan Tiwari A, Shukla M. (2020) The relationship between self-esteem and self-forgiveness: understanding the mediating role of positive and negative self-compassion. 10.22541/au.158981530.01103201.

[20] Yao S, Chen J, Yu X, Sang J (2017). Mediator roles of interpersonal forgiveness and self-forgiveness between self-esteem and subjective well-being. Curr Psychol.

[21] Hill PL, Allemand M (2010). Forgivingness and adult patterns of individual differences in environmental mastery and personal growth. J Res Pers.

[22] Strelan P (2007). The prosocial, adaptive qualities of just world beliefs: implications for the relationship between justice and forgiveness. Pers Individ Dif.

[23] Holmgren MR. (1998) Self-Forgiveness and Responsible Moral Agency.

[24] Haidt J (2001). The emotional dog and its rational tail: a Social Intuitionist Approach to Moral Judgment. Psychol Re\itA.

[25] Leary MR (2007). Motivational and emotional aspects of the self. Annu Rev Psychol.

[26] DeWall CN, Twenge JM, Koole SL, Baumeister RF, Marquez A, Reid MW (2011). Automatic emotion Regulation after Social Exclusion: tuning to positivity. Emotion.

[27] Wenzel M, Woodyatt L, Hedrick K (2012). No genuine self-forgiveness without accepting responsibility: Value reaffirmation as a key to maintaining positive self-regard. Eur J Soc Psychol.

[28] woodyAtt lydiA weNZel miCHAel. Self-forgiveness and restoration WOODYATT and WENZEL self-forGIveness and restoratIon of an offender followInG. an Interpersonal transGressIon; 2013.

[29] Suzuki M, Jenkins T (2022). The role of (self-)forgiveness in restorative justice: linking restorative justice to desistance. Eur J Criminol.

[30] Woodyatt L, Wenzel M (2013). The psychological immune response in the face of transgressions: Pseudo self-forgiveness and threat to belonging. J Exp Soc Psychol.

[31] Krentzman AR, Webb JR, Jester JM, Harris JI (2018). Longitudinal relationship between forgiveness of self and forgiveness of others among individuals with alcohol use disorders. Psycholog Relig Spiritual.

[32] Webb JR, Bumgarner DJ, Conway-Williams E, Dangel T, Hall BB (2017). A consensus definition of self-forgiveness: implications for assessment and treatment. Spiritual Clin Pract.

[33] Bauer L, Duffy J, Fountain E, Halling S, Holzer M, Jones E, Leifer M, Rowe JO. (1992) Exploring Self-Forgiveness. 10.1007/BF00986793 24272882

[34] Terzino KA. Self-forgiveness for interpersonal and intrapersonal transgressions.

[35] Toussaint L, Barry M, Bornfriend L, Markman M (2014). Restore: the Journey toward Self-Forgiveness: a Randomized Trial of Patient Education on Self-Forgiveness in Cancer patients and caregivers. J Health Care Chaplain.

[36] Ghavami T, Kazeminia M, Rajati F (2022). The effect of lavender on stress in individuals: a systematic review and meta-analysis. Complement Ther Med.

[37] Page MJ, McKenzie JE, Bossuyt PM (2021). The PRISMA 2020 statement: an updated guideline for reporting systematic reviews. PLoS Med.

[38] Martins Esteves I, Coelho MS. EEectiveness of family-centred educational interventions for anxiety, pain and behaviours of children and adolescents and anxiety of their parents during the perioperative journey: a systematic review and meta-analysis.

[39] Lockwood C, Munn Z, Porritt K (2015). Qualitative research synthesis: methodological guidance for systematic reviewers utilizing meta-aggregation. Int J Evid Based Healthc.

[40] Lander A (2012). Toward the incorporation of forgiveness therapy in healing the wounds of eating disorders: a case study in self-forgiveness. Clin Case Stud.

[41] Scherer M, Worthington EL, Hook JN, Campana KL (2011). Forgiveness and the bottle: promoting self-forgiveness in individuals who abuse alcohol. J Addict Dis.

[42] Bell CM, Davis DE, Griffin BJ, Ashby JS, Rice KG (2017). The promotion of self-forgiveness, responsibility, and willingness to make reparations through a workbook intervention. J Posit Psychol.

[43] Campana K, SELF-FORGIVENESS INTERVENTIONS FOR WOMEN SELF-FORGIVENESS INTERVENTIONS. FOR WOMEN EXPERIENCING A BREAKUP EXPERIENCING A BREAKUP.

[44] Coyle CT, Enright RD. (1997) Forgiveness Intervention With Postabortion Men. 10.1037//0022-006x.65.6.1042 9420366

[45] Eaton KW, Ferrari TM (2020). Heart Rate Variability during an Internal Family systems Approach to Self-Forgiveness. Int J Clin Exp Physiol.

[46] Exline JJ, Root BL, Yadavalli S, Martin AM, Fisher ML (2011). Reparative behaviors and Self-forgiveness: effects of a laboratory-based Exercise. Self Identity.

[47] Hanna W. Scholarship at UWindsor Scholarship at UWindsor benefits of Self-Forgiveness on Well-Bieng and Self-Forgiveness benefits of Self-Forgiveness on Well-Bieng and Self-. Forgiveness Facilitating Factors Facilitating Factors; 2012.

[48] Jo KH, An GJ (2018). Effects of a group reminiscence program on self-forgiveness, life satisfaction, and death anxiety among institutionalized older adults. Korean J Adult Nurs.

[49] Franz Y, Kahija L, Rahmandani A, Salma S, THE EFFECTIVENESS. OF FORGIVENESS MEDITATION INTERVENTION IN THE GROUP OF EMERGING ADULT STUDENTS.

[50] Maguen S, Burkman K, Madden E, Dinh J, Bosch J, Keyser J, Schmitz M, Neylan TC (2017). Impact of killing in War: a Randomized, Controlled Pilot Trial. J Clin Psychol.

[51] Massengale MA. Randomized Control Trial adapting a self-forgiveness A Randomized Control Trial adapting a self-forgiveness intervention for perfectionists intervention for perfectionists. 10.57709/18714397.

[52] Maynard PG, van Kessel K, Feather JS (2023). Self-forgiveness, self-compassion and psychological health: a qualitative exploration of change during compassion focused therapy groups. Psychol Psychotherapy: Theory Res Pract.

[53] Ogunyemi D, Sugiyama NI, Ferrari TM (2020). A Professional Development Workshop to Facilitate Self-Forgiveness. J Grad Med Educ.

[54] Parlak S, Oksuz Gul F (2021). Psychodrama oriented group therapy for forgiveness in university students. Arts Psychother.

[55] Peterson SJ, Van Tongeren DR, Womack SD, Hook JN, Davis DE, Griffin BJ (2017). The benefits of self-forgiveness on mental health: evidence from correlational and experimental research. J Posit Psychol.

[56] Woodyatt L, Wenzel M (2014). A needs-based perspective on self-forgiveness: addressing threat to moral identity as a means of encouraging interpersonal and intrapersonal restoration. J Exp Soc Psychol.

[57] Záhorcová L, Enright R, Halama P (2023). The effectiveness of a Forgiveness Intervention on Mental Health in Bereaved Parents—A Pilot Study. Omega (United States).

[58] La Kahija YF, Rahmandani A, Salma S (2022). The effectiveness of forgiveness meditation intervention in the Group of emerging adult students. Jurnal Psikologi.

[59] Worthington EL. (2013) Moving Forward: six steps to forgiving yourself and breaking free from the Past Self-Directed Learning Workbook an intervention designed to Promote Self-Forgiveness.

[60] THE ART. AND SCIENCE OF FORGIVING.

[61] Maltby J, Macaskill A, Day L. Failure to forgive self and others: a replication and extension of the relationship between forgiveness, personality, social desirability and general health.

[62] Dearing RL, Stuewig J, Tangney JP (2005). On the importance of distinguishing shame from guilt: relations to problematic alcohol and drug use. Addict Behav.

[63] Lundahl BW, Taylor MJ, Stevenson R, Roberts KD (2008). Process-based forgiveness interventions: a meta-analytic review. Res Soc Work Pract.

[64] Toussaint L, Worthington EL, Cheadle A, Marigoudar S, Kamble S, Büssing A (2020). Efficacy of the REACH Forgiveness Intervention in Indian College Students. Front Psychol.

[65] Baskin TW, Enright RD (2004). Intervention studies on forgiveness: a Meta-analysis. J Couns Dev.

[66] Akhtar S, Barlow J (2018). Forgiveness therapy for the Promotion of Mental Well-Being: a systematic review and Meta-analysis. Trauma Violence Abuse.

[67] The number of. psychosocial interventions for relatives of adults with serious.

[68] Greenberg LS, Warwar SH, Malcolm WM (2008). Differential effects of emotion-focused therapy and psychoeducation in facilitating forgiveness and letting go of emotional injuries. J Couns Psychol.

[69] Schwartz RC (2013). Moving from acceptance toward transformation with internal family systems therapy (IFS). J Clin Psychol.

[70] Hall JH, Fincham FD. (2008) HALL AND FINCHAM SELF-FORGIVENESS THE TEMPORAL COURSE OF SELF-FORGIVENESS.

[71] Westerhof GJ, Bohlmeijer ET (2014). Celebrating fifty years of research and applications in reminiscence and life review: state of the art and new directions. J Aging Stud.

[72] Mudgal S, Tiwari GK. Self-Forgiveness and Life Satisfaction in People Living with HIV/AIDS.

[73] Dipietro EK, Unforgiving Pain. A Qualitative Exploration of Chronic Pain and Unforgiving Pain: A Qualitative Exploration of Chronic Pain and Self-Forgiveness Self-Forgiveness.

[74] Arslan G, Coşkun M (2022). Social Exclusion, Self-Forgiveness, Mindfulness, and internet addiction in College students: a Moderated Mediation Approach. Int J Ment Health Addict.

[75] Wohl MJA, Thompson A (2011). A dark side to self-forgiveness: forgiving the self and its association with chronic unhealthy behaviour. Br J Soc Psychol.

[76] Onody AP, Woodyatt L, Wenzel M, Cibich M, Sheldon A, Cornish MA (2020). Humility and its relationship to Self-condemnation, defensiveness and self-forgiveness following interpersonal transgressions. J Psychol Theol.

[77] Tiwari GK, Pandey R, Parihar P, Rai PK. (2020) Understanding the mediating role of self-esteem between the relationship of self-forgiveness and human flourishing. 10.22541/au.158981525.55950259.

