# Supplementary material for: Psychological interventions to promote self-forgiveness: a systematic review
Source: BMC Psychol. 2024 May 9;12:258. doi: 10.1186/s40359-024-01671-3 (PMC11084121; doi:10.1186/s40359-024-01671-3)
Supplement: Supplementary file 1 — Supplementary Material 1 [file 40359_2024_1671_MOESM1_ESM.docx]

**Supplement 1 Quality assessment results of randomized controlled trials using JBI critical appraisal Checklist**

| **Author(s) & Year** | **Q1** | **Q2** | **Q3** | **Q4** | **Q5** | **Q6** | **Q7** | **Q8** | **Q9** | **Q10** | **Q11** | **Q12** | **Q13** | **Quality score** |
| --- | --- | --- | --- | --- | --- | --- | --- | --- | --- | --- | --- | --- | --- | --- |
| Bell et al. (2017) | N | N | Y | Y | N | U | Y | N | Y | Y | Y | Y | Y | 8 |
| Campana (2010) | Y | Y | Y | Y | N | U | Y | Y | Y | Y | Y | Y | Y | 11 |
| Cornish et al. (2015) | Y | Y | Y | Y | U | Y | Y | Y | Y | Y | Y | Y | Y | 12 |
| Coyle & Enright (1997) | Y | N | Y | Y | N | U | Y | Y | Y | Y | Y | Y | Y | 10 |
| Exline et al. (2011) | N | N | Y | Y | U | N | Y | Y | Y | Y | Y | Y | Y | 9 |
| Griffin et al. (2015) | N | N | Y | Y | U | Y | Y | Y | Y | Y | Y | Y | Y | 10 |
| Hanna (2012) | Y | Y | Y | Y | N | U | Y | Y | Y | Y | Y | Y | Y | 11 |
| Maguen (2017) | Y | Y | Y | N | Y | U | U | Y | Y | Y | Y | Y | Y | 10 |
| Massengale,M. et al. (2020) | Y | Y | Y | Y | N | U | Y | N | Y | Y | Y | Y | Y | 10 |
| Peterson et al. (2016) | N | N | Y | Y | U | U | Y | Y | Y | Y | Y | Y | Y | 9 |
| Scherer et al. (2011) | N | N | Y | Y | U | U | Y | Y | Y | Y | Y | Y | Y | 9 |
| Toussaint et al. (2014) | N | N | Y | Y | U | U | Y | N | Y | Y | Y | Y | Y | 8 |
| Woodyatt & Wenzel (2014) | Y | Y | Y | Y | Y | U | Y | Y | Y | Y | Y | Y | Y | 12 |
| Zahorcova et al. (2021) | Y | N | Y | Y | N | Y | Y | Y | Y | Y | Y | Y | Y | 11 |

Q1 – Q13 indicates questions 1 to 13 as per the JBI appraisal tool.

Y – Yes, N – No, U – Unclear N/A – Not/Applicable

**Supplement 1 Quality assessment results of quasi-experimental studies using JBI critical appraisal Checklist**

| **Author(s) & Year** | **Q1** | **Q2** | **Q3** | **Q4** | **Q5** | **Q6** | **Q7** | **Q8** | **Q9** | **Quality score** |
| --- | --- | --- | --- | --- | --- | --- | --- | --- | --- | --- |
| Eaton & Ferrari (2020) | Y | Y | NA | NA | Y | NA | Y | Y | Y | 6 |
| Jo & An (2018) | Y | Y | N | Y | Y | N | Y | Y | Y | 7 |
| Kahija, Y.F.L., et al. (2022) | Y | Y | N | N | Y | Y | Y | Y | Y | 7 |
| Ogunyemi et al. (2020) | Y | Y | N | N | Y | N | N | Y | Y | 5 |
| Parlak & Gul. (2021) | Y | Y | N | Y | Y | Y | N | Y | Y | 7 |

Q1 – Q9 indicates questions 1 to 9 as per the JBI appraisal tool.

Y – Yes, N – No, U – Unclear N/A – Not/Applicable

**Supplement 1 Quality assessment results of Qualitative studies using JBI critical appraisal Checklist**

| **Author(s) & Year** | **Q1** | **Q2** | **Q3** | **Q4** | **Q5** | **Q6** | **Q7** | **Q8** | **Q9** | **Q10** | **Quality score** |
| --- | --- | --- | --- | --- | --- | --- | --- | --- | --- | --- | --- |
| Lander (2012) | Y | Y | Y | Y | Y | U | Y | Y | N | Y | 8 |
| Maynard et al. (2023) | Y | Y | Y | Y | Y | Y | U | N | Y | Y | 8 |

Q1 – Q10 indicates questions 1 to 10 as per the JBI appraisal tool.

Y - Yes, N – No, U – Unclear N/A – Not/Applicable
